# Effect of Pediatric Heart Transplant Waitlist Inactivation on Waitlist and Posttransplant Outcomes

**DOI:** 10.1111/petr.70454

**Published:** 2026-09-15

**Authors:** Dean S. Karahalios, Christina Laternser, Philip Thrush, Adam Morrison, Michael Mongé, Defne A. Magnetta

**Affiliations:** ^1^ Division of Pediatric Cardiology University of Wisconsin‐Madison American Family Children's Hospital Madison Wisconsin USA; ^2^ Division of Pediatric Cardiology Northwestern University Ann and Robert H. Lurie Children's Hospital of Chicago Chicago Illinois USA; ^3^ Center for Cardiovascular Innovation Ann and Robert H. Lurie Children's Hospital of Chicago Chicago Illinois USA; ^4^ Division of Cardiovascular Surgery Northwestern University Ann and Robert H. Lurie Children's Hospital of Chicago Chicago Illinois USA

**Keywords:** pediatric heart transplant, short‐term posttransplant survival, Status 7, waitlist inactivation, waitlist mortality

## Abstract

**Background:**

Little is known about pediatric heart transplant candidates facing waitlist inactivation. We characterized them and assessed the impact of inactivation on waitlist and transplant outcomes.

**Methods:**

The Organ Procurement and Transplantation Network database was queried for pediatric heart‐only transplant candidates. Characteristics, waitlist mortality, and 1‐year posttransplant survival between inactivated and non‐inactivated patients were compared. Predicted probability for the composite outcome of waitlist mortality or removal due to clinical deterioration over time was computed.

**Results:**

Of 5202 patients, 1716 (33%) were inactivated, mostly due to temporary illness (58%). Reasons for inactivation varied by age but not diagnosis. Inactivated patients had longer waitlist duration (171 [77–391] vs. 62 [21–144], *p* < 0.001), more congenital heart disease (61.0% vs. 55.2%, *p* < 0.001), and were more often supported by ventricular assist device (47.9% vs. 32.9%, *p* < 0.001) and invasive ventilation at listing (19.9% vs. 13.6%, *p* < 0.001). Inactivated patients were less likely listed Status 1A (56.8% vs. 62.3%, *p* < 0.001) and less likely under intensive care at transplant (43.9% vs. 50.2%, *p* < 0.001). Inactivated patients had higher 1‐year waitlist mortality and removal due to clinical deterioration (SHR 4.40 [3.72–5.20], *p* < 0.001), though this risk decreased with longer inactive time. One‐year posttransplant survival was similar between groups.

**Conclusions:**

Waitlist inactivation was strongly associated with 1‐year waitlist mortality and removal due to clinical deterioration. However, this risk decreased with longer inactive time and made no difference in short‐term posttransplant survival. Thus, the prognostic implications of inactivation are dynamic and can inform future donor allocation policy development.

## Graphical abstract

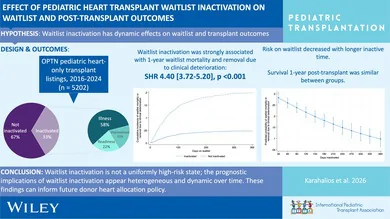

**Abbreviations**
BiVAD: biventricular assist device
CHD: congenital heart disease
ECMO: extracorporeal membrane oxygenation
ICU: intensive care unit
IQR: interquartile range
LVAD: left ventricular assist device
OPTN: Organ Procurement and Transplantation Network
RVAD: right ventricular assist device
TAH: total artificial heart
VAD: ventricular assist device

## 1 Introduction

After listing for pediatric heart transplant, patients can be temporarily inactivated on the waitlist due to various factors that would affect eligibility or success of the heart transplant [1]. Examples of such temporary factors can include a temporary comorbidity such as infection, the development of surgical risk factors such as obesity, travel far away from the listing center, poor adherence to the current medical regimen, and a change in insurance status, among other factors. Reactivation on the waitlist and eventual transplant then depends on addressing specific patient factors leading to the inactivation.

Waitlist inactivation is common among certain adult heart transplant candidates, occurring in approximately 18% of candidates listed Status 1 or Status 2 and portends higher posttransplant mortality [2]. However, the use of waitlist inactivation in the current era of pediatric heart allocation has not yet been assessed. Furthermore, the impact of waitlist inactivation on pre‐ and posttransplant outcomes is unclear. Studying the impact of temporary waitlist inactivation on waitlist and transplant outcomes is valuable due to the potential to inform listing strategies and future iterations of the pediatric donor heart allocation system.

## 2 Methods

### 2.1 Patient Selection

This retrospective database study was exempt from review by our Institutional Review Board. The Organ Procurement and Transplantation Network (OPTN) database was queried for pediatric heart‐only transplant candidates under 18 years of age. Patients listed before the updated pediatric heart allocation policy revision on March 22, 2016 were excluded. Patients listed for retransplant and patients with multiple listings were also excluded. Patients were followed until 1 year after transplant or the day of the last observation, which was June 30, 2024.

### 2.2 Statistical Analysis

Categorical variables were reported as frequency and percentage, while continuous variables were reported as median with interquartile range (IQR). Chi square, Fisher's exact, Wilcoxon rank sum, and Kruskal–Wallis tests compared characteristics between transplant candidates and recipients by history of inactivation, by reason for inactivation, by listing region, and by transplant center volume as appropriate. For analyses of age, three categories postulated to have different patterns of inactivation were defined: infants, prepubertal children, and postpubertal adolescents. For analyses by transplant center volume, the number of transplant listings of each anonymized transplant center was averaged over the 8‐year study period. Centers with an average transplant volume of > 15 transplants per year were categorized as high volume; centers with an average of 5–15 transplants per year were categorized as medium volume; and centers with an average of < 5 transplants per year were categorized as low volume.

To study waitlist outcomes of the cohort, unadjusted cumulative incidence functions were estimated for the events of transplantation, waitlist removal due to death or clinical deterioration, and waitlist removal for other reasons. Alternative terminal waitlist outcomes were treated as competing events. The probability of remaining on the waitlist was estimated as the event‐free survival probability for any terminal waitlist outcome. Follow‐up was administratively censored at 365 days. Estimates were displayed for the overall cohort and stratified by reason for inactivation.

Fine‐Gray competing risks regression was used to assess the composite outcome of waitlist mortality or removal due to clinical deterioration in inactivated and non‐inactivated patients. Multivariable Fine‐Gray competing risks regression measured the association between inactivation and the same composite outcome, accounting for covariates. Logistic regression with estimated marginal means was then employed to predict the composite outcome according to time inactivated on the waitlist. This composite outcome of waitlist mortality or removal due to clinical deterioration was chosen to better capture the notion of waitlist failure as a lost opportunity to achieve transplant, to neutralize center‐level variation in waitlist removal practices, and to avoid informative censoring bias related to waitlist removals.

Lastly, Kaplan–Meier analysis evaluated posttransplant survival at 1 year between inactivated and non‐inactivated patients. Statistical analysis was performed using Stata (StataNow‐SE version 19.5; StataCorp LLC, College Station, Texas, USA). Significance level was set at 0.05.

## 3 Results

### 3.1 Profile of Inactivated Patients

In total, 5202 patients met inclusion criteria. Of these, 1716 (33%) were inactivated. The duration of inactive time among those inactivated was heavily skewed with a median of 25 (7–123) days and a range of 2839 days. Those listed as Status 1A had the shortest inactive time, and those listed as Status 2 had the longest inactive time (17 [6–63] days for Status 1A; 29 [9–142] days for Status 1B; 84 [16–331] days for Status 2, *p* < 0.001).

**FIGURE 1:**
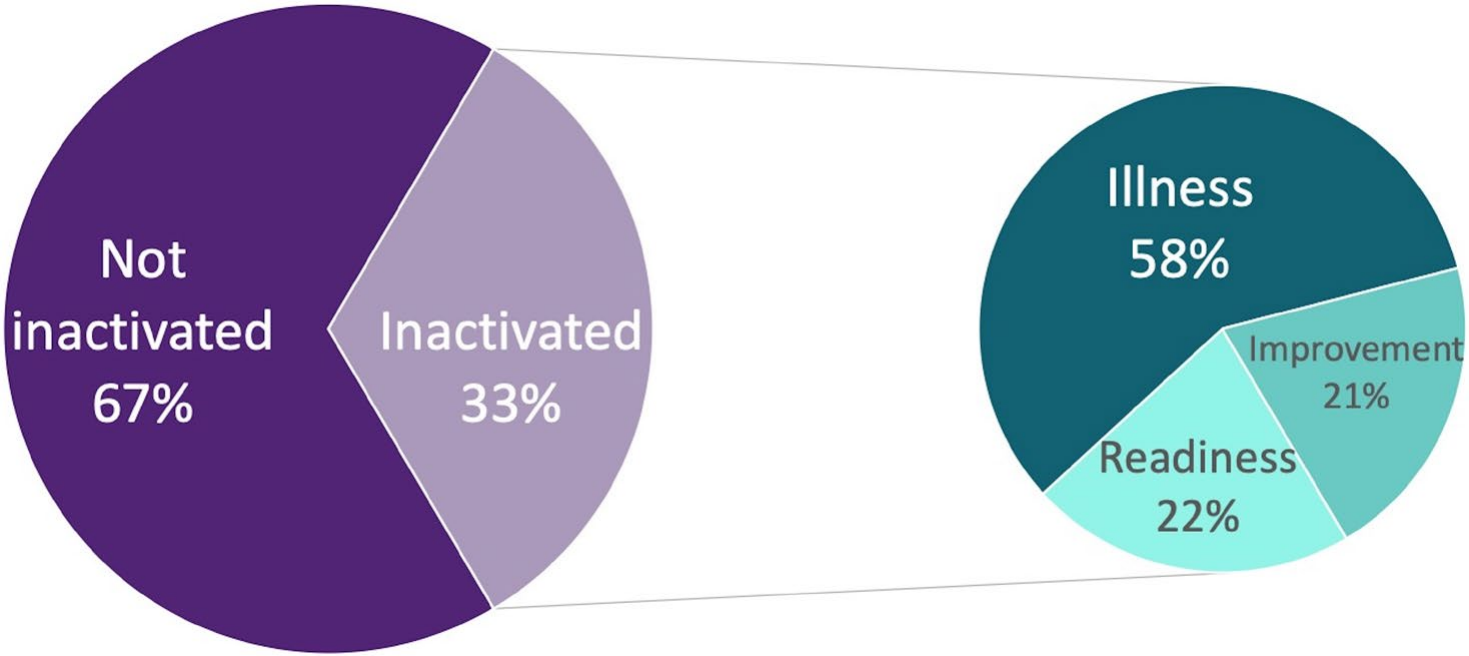
Frequency and reasons for waitlist inactivation.

Figure 1 outlines the frequency of inactivation and reasons for inactivation consolidated into three categories: temporary illness, clinical improvement, and patient or program readiness for transplant. In all, 992 (58%) patients were inactivated due to illness, 352 (21%) due to clinical improvement, and 372 (22%) due to patient or program readiness issues such as travel or staffing, among other reasons.

Table 1 outlines patient characteristics by reasons for waitlist inactivation. Overall, reasons for inactivation differed by age. Infants < 1 year of age were inactivated more commonly due to temporary illness (48.4%) or clinical improvement (58.0%) versus readiness issues (16.7%, *p* < 0.001). Conversely, older patients aged 10–18 years were inactivated more commonly due to readiness issues (46.2%) and less often due to temporary illness (22.5%) or clinical improvement (15.6%, *p* < 0.001). This relationship persisted when age was compared as a continuous variable. The median age for patients inactivated for readiness issues was highest at 8 (2–14) years. The median age was less at 1 (0–8) years for those inactivated for illness and 0 (0–4) years for those inactivated for improvement (*p* < 0.001).

**TABLE 1:** Patient characteristics and reasons for waitlist inactivation.

*n* (%) or median (IQR)	Illness (*n* = 992)	Improvement (*n* = 352)	Readiness (*n* = 372)	*p*
Age at listing (years)	1 (0–8)	0 (0–4)	8 (2–14)	< 0.001
< 1	480 (48.4%)	204 (58.0%)	62 (16.7%)	< 0.001
1–9	289 (29.1%)	93 (26.4%)	138 (37.1%)	
10–17	223 (22.5%)	55 (15.6%)	172 (46.2%)	
Diagnosis				
Cardiomyopathy	344 (34.7%)	146 (23.0%)	146 (39.3%)	0.050
CHD	625 (63.0%)	198 (56.3%)	223 (60.0%)	
Other	23 (2.3%)	8 (2.3%)	3 (0.8%)	
Inactivated time (days)	14 (5–36)	251 (95–565)	28 (8–125)	< 0.001

On the other hand, there was no statistically significant association between reason for waitlist inactivation and diagnosis. Transplant candidates who were inactivated due to clinical improvement had the most inactive time (251 [95–565] days for clinical improvement vs. 14 [5–36] days for illness and 28 [8–125] days for readiness, *p* < 0.001).

Tables S1 and S2 show patterns of inactivation by OPTN listing region and transplant center volume, respectively (see Supporting Information). Inactivation was most common in Region 10 (44.9%, *p* < 0.001) and at high volume centers (22.8%, *p* = 0.024). The relative proportion of each reason for inactivation among regions generally mirrored those of the entire cohort, but low volume centers inactivated patients most commonly due to illness (77.1% at low volume centers vs. 63.7% at medium volume centers vs. 73.4% at high volume centers, *p* = 0.015). Region 4 and low volume centers had the most listings initially listed as Status 7 (2.9%, *p* = 0.008; and 2.0%, *p* = 0.038, respectively). There was no significant variation in total inactive time by region or by transplant center volume.

### 3.2 Comparison of Inactivated vs. Non‐Inactivated Patients

Table 2 summarizes listing and waitlist characteristics of inactivated and non‐inactivated patients. Inactivated patients were younger (1 [0–10] vs. 5 [0–13] years, *p* < 0.001). Infants under 1 year of age comprised 43.5% of inactivated patients versus 30.6% of non‐inactivated patients (*p* < 0.001). Inactivated patients had more congenital heart disease (61.0% vs. 55.2%, *p* < 0.001) and were more likely to be supported by any type of ventricular assist device (VAD, 47.9% vs. 32.9%, *p* < 0.001). Left ventricular assist device (LVAD) was the most common type of device employed (39.8% vs. 27.6%, *p* < 0.001). Invasive ventilation at the time of listing was more common among inactivated patients (19.9% vs. 13.6%, *p* < 0.001). Pretransplant infection was also more common among the inactivated cohort (17.9% vs. 12.5%, *p* = 0.001).

**TABLE 2:** Listing and waitlist characteristics of inactivated and non‐inactivated patients.

*n* (%) or median (IQR)	Inactivated (*n* = 1716)	Not inactivated (*n* = 3486)	*p*
Age at listing (years)	1 (0–10)	5 (0–13)	< 0.001
< 1	746 (43.5%)	1068 (30.6%)	< 0.001
1–9	520 (30.3%)	1037 (29.8%)	
10–17	450 (26.2%)	1381 (39.6%)	
Diagnosis			
Cardiomyopathy	636 (37.1%)	1477 (42.4%)	< 0.001
CHD	1046 (61.0%)	1923 (55.2%)	
Other	34 (2.0%)	86 (2.5%)	
Status at listing			
1A	974 (56.8%)	2173 (62.3%)	< 0.001
1B	340 (19.8%)	765 (21.9%)	
2	331 (19.3%)	548 (15.7%)	
7	71 (4.1%)	0 (0.0%)	
VAD(s) at listing			
None	377 (52.1%)	1920 (67.1%)	< 0.001
LVAD	288 (39.8%)	790 (27.6%)	
RVAD	17 (2.4%)	38 (1.3%)	
TAH	2 (0.3%)	6 (0.2%)	
BiVAD	39 (5.4%)	109 (3.8%)	
Invasive ventilation at listing	342 (19.9%)	473 (13.6%)	< 0.001
Pretransplant infection	129 (17.9%)	358 (12.5%)	0.001
Total waitlist duration (days)	171 (77–391)	62 (21–144)	< 0.001
Waitlist mortality	244 (14.2%)	155 (4.5%)	< 0.001
Cause of death on waitlist			
Infection	11 (4.5%)	5 (3.2%)	0.006
Cardiovascular	100 (41.0%)	88 (56.8%)	
Neurologic	28 (11.5%)	7 (4.5%)	
Hemorrhage	10 (4.1%)	9 (5.8%)	
Multiorgan failure	60 (24.6%)	22 (14.2%)	
Other/unknown	35 (14.3%)	24 (15.5%)	

On the other hand, inactivated patients were less likely to be listed initially as Status 1A (56.8% vs. 62.3%, *p* < 0.001). Seventy‐one (4.1%) inactivated patients were initially listed inactivated as Status 7. Of these 71 patients inactivated at listing, 21.1% were 17 years old and 18.3% were < 1 year old. The most common reason for inactivation among 17‐year‐old patients initially listed as Status 7 was readiness (60.0% vs. 33.3% for illness and 6.7% for clinical improvement, *p* = 0.011). Forty (56.3%) patients listed as Status 7 were supported by either extracorporeal membrane oxygenation (ECMO) or VAD at the time of listing, though there was no correlation between mechanical circulatory support use at listing and age at listing (median age 12 [3–15] years for those with support vs. 10 [1–17] years for those without support, *p* = 0.824).

Total waitlist duration was significantly longer for inactivated patients (171 [77–391] vs. 62 [21–144] days, *p* < 0.001) and their waitlist mortality exceeded that of non‐inactivated patients (14.2% vs. 4.5%, *p* < 0.001). Regarding cause of death on the waitlist, the inactivated cohort was less likely to die from cardiovascular causes (41.0% vs. 56.8%) but was more likely to die from extracardiac causes including infection (4.5% vs. 3.2%), neurologic causes (11.5% vs. 4.5%), and multiorgan failure (24.6% vs. 14.2%). On the other hand, non‐inactivated patients were more likely to experience waitlist mortality due to hemorrhage (5.8% vs. 4.1%, *p* = 0.006).

Table 3 summarizes characteristics of transplant recipients with and without a history of inactivation. Transplant recipients with a history of inactivation tended to be transplanted at a categorical age > 9 years (44.4% vs. 39.9%, *p* = 0.015); however, this association did not persist when age was analyzed as a continuous variable. Transplant recipients with a history of inactivation were also more commonly bridged to transplant with any type of VAD (47.9% vs. 32.9%, *p* < 0.001) and were more commonly in the outpatient setting at the time of transplant (27.0% vs. 20.4%, *p* < 0.001). Transplant recipients with a history of inactivation had longer total waitlist duration than those without a history of inactivation (160 [88–281] vs. 58 [19–134] days, *p* < 0.001). There were no statistically significant differences in diagnosis, listing status at the time of transplant, or invasive ventilation at the time of transplant.

**TABLE 3:** Characteristics of transplant recipients with and without history of inactivation.

*n* (%) or median (IQR)	Inactivated (*n* = 740)	Not inactivated (*n* = 2926)	*p*
Age at transplant (years)	5 (1–14)	7 (0–14)	0.236
< 1	752 (25.7%)	184 (24.9%)	0.015
1–9	875 (29.9%)	261 (35.3%)	
10–17	1299 (44.4%)	295 (39.9%)	
Diagnosis			
Cardiomyopathy	331 (44.7%)	1330 (45.5%)	0.095
CHD	401 (54.2%)	1529 (52.3%)	
Other	8 (1.1%)	67 (2.3%)	
Status at transplant			
1A	611 (82.6%)	2381 (81.4%)	0.647
1B	107 (14.5%)	463 (15.8%)	
2	22 (3.0%)	82 (2.8%)	
VAD(s) at transplant			
None	377 (52.1%)	1920 (67.1%)	< 0.001
LVAD	288 (39.8%)	790 (27.6%)	
RVAD	17 (2.4%)	38 (1.3%)	
TAH	2 (0.3%)	6 (0.2%)	
BiVAD	39 (5.4%)	109 (3.8%)	
Invasive ventilation at transplant	79 (10.7%)	322 (11.0%)	0.798
Location at transplant			
ICU	317 (43.9%)	1439 (50.2%)	< 0.001
Acute care	211 (29.2%)	841 (29.4%)	
Not hospitalized	195 (27.0%)	585 (20.4%)	
Total waitlist duration (days)	160 (88–281)	58 (19–134)	< 0.001

### 3.3 Waitlist Outcomes

**FIGURE 2:**
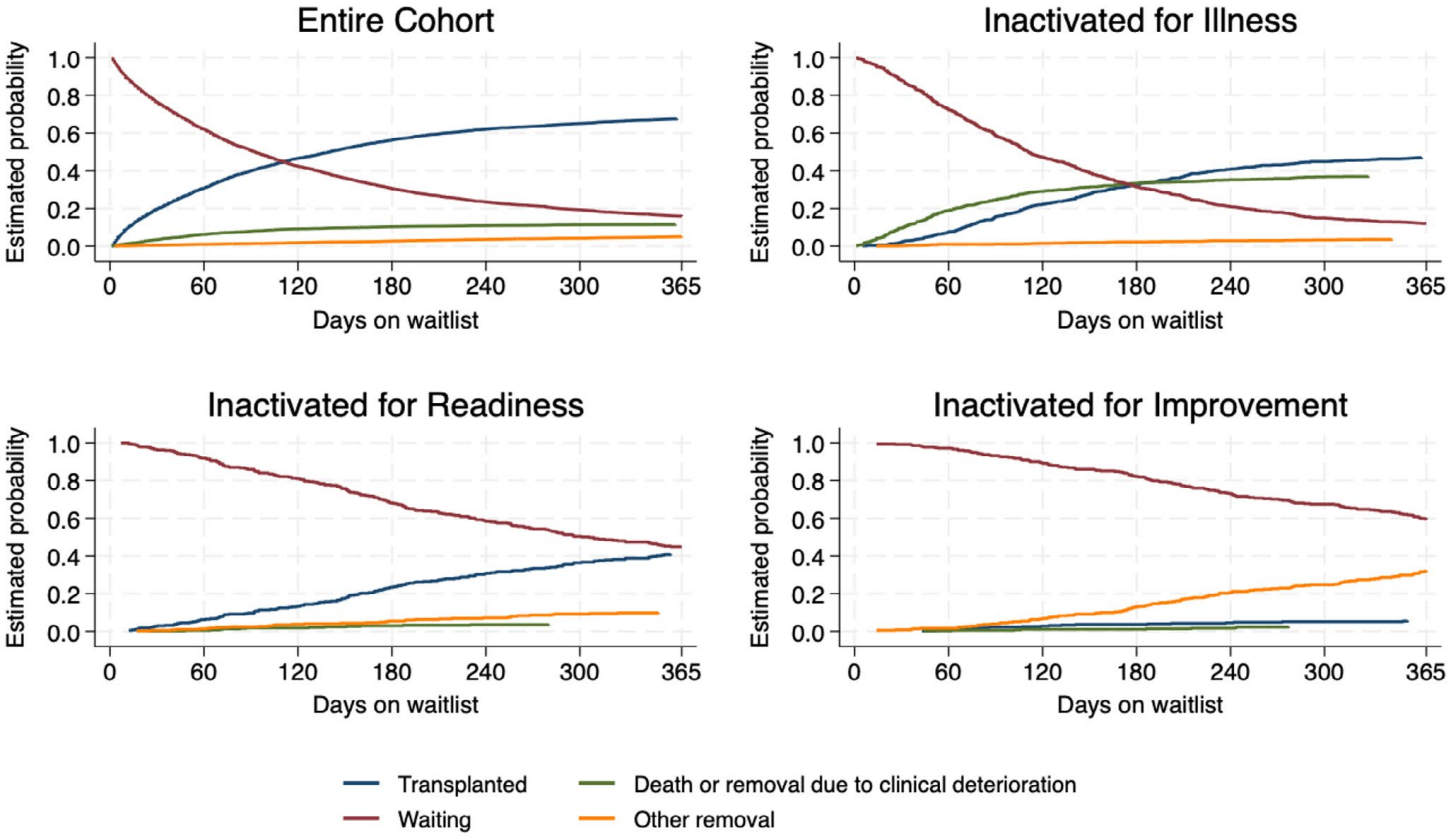
Waitlist outcomes for the entire patient cohort and the inactivated cohort stratified by reason for inactivation.

Figure 2 compares waitlist outcomes of the entire patient cohort to those inactivated for each specific inactivation category, while Table 4 numerically describes the outcomes of each group. Among the entire patient cohort of 5202 patients, 3675 (70.7%) achieved transplant, while 628 (12.1%) died or were removed from the waitlist for clinical deterioration. Meanwhile, 423 (8.1%) of the patients were removed from the waitlist for other reasons and 476 (9.2%) remained on the waitlist.

**TABLE 4:** Waitlist disposition of all patients as well as patients inactivated for each reason for inactivation.

*n* (%)	All patients (*n* = 5202)	Inactivated patients (*n* = 1716)			
		Inactivated for illness (*n* = 992)	Inactivated for improvement (*n* = 352)	Inactivated for readiness (*n* = 372)	*p*
Transplanted	3675 (70.7%)	506 (51.0%)	31 (8.8%)	207 (55.7%)	< 0.001
Death or removal due to clinical deterioration	628 (12.1%)	380 (38.3%)	17 (4.8%)	19 (5.1%)	
Other removal	423 (8.1%)	49 (4.9%)	222 (63.1%)	70 (18.8%)	
Waiting	476 (9.2%)	57 (5.8%)	82 (23.3%)	76 (20.4%)	

Among the inactivated cohort, those inactivated due to illness experienced the highest proportion of waitlist mortality or removal due to clinical deterioration (38.3% for illness vs. 4.8% for improvement and 5.1% for readiness). Patients inactivated due to clinical improvement or readiness had high rates of remaining on the waitlist (23.3% and 20.4%, respectively, vs. 5.8% for illness). Those inactivated for illness and readiness achieved the highest rates of transplant (51.0% and 55.7%, respectively, vs. 8.8% for improvement, *p* < 0.001).

**FIGURE 3:**
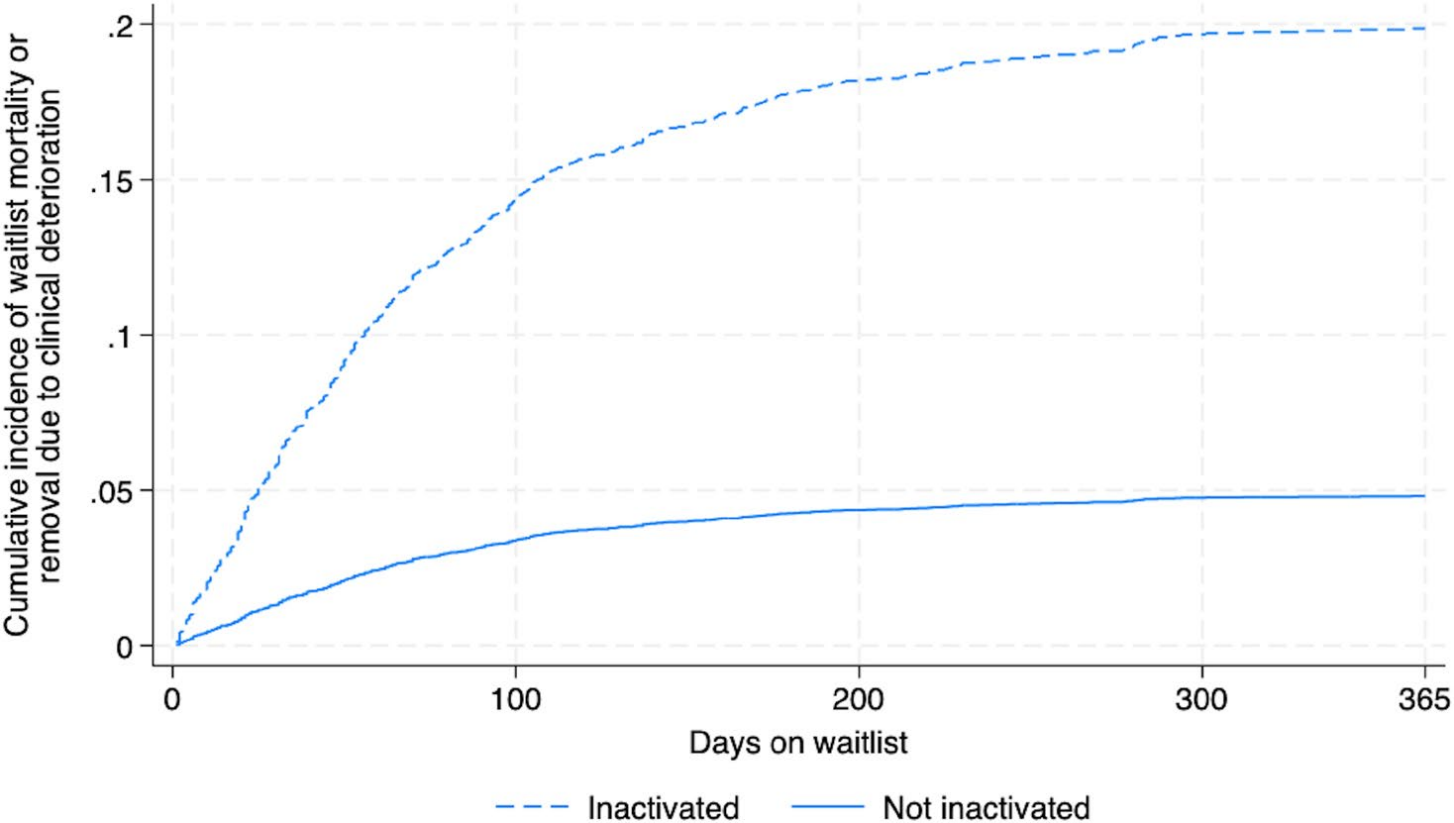
Fine‐Gray competing risks regression for the composite outcome of waitlist mortality or removal due to clinical deterioration among inactivated and non‐inactivated patients.

Fine‐Gray competing risks regression compared the composite outcome of waitlist mortality or removal due to clinical deterioration according to history of waitlist inactivation (Figure 3). Inactivated patients experienced much higher rates of the composite outcome than non‐inactivated patients, particularly within the first 100 days on the waitlist (SHR 4.40 [3.72–5.20], *p* < 0.001). Subsequently, the risk of the composite outcome was generally equal between groups.

Multivariable Fine‐Gray competing risks regression was also used to compare the relative strength of the association between waitlist inactivation and the composite outcome (Table 5). Broken down by reason for inactivation with readiness as a reference, there was no significant difference in the risk for the composite outcome with inactivation due to improvement (SHR 0.74 [0.38–1.41], *p* = 0.355). However, inactivation due to illness was much more strongly associated with death or deterioration on the waitlist (SHR 6.85 [4.31–10.88], *p* < 0.001).

**TABLE 5:** Multivariable Fine‐Gray competing risks regression for the composite outcome of waitlist mortality or removal due to clinical deterioration.

	Subdistribution hazard ratio (95% CI)	*p*
Inactivation reason		
Readiness	Reference	
Illness	6.85 (4.31–10.88)	< 0.001
Improvement	0.74 (0.38–1.41)	0.355
Diagnosis		
Cardiomyopathy	Reference	
CHD	1.86 (1.46–2.36)	< 0.001
Other	3.98 (2.09–7.56)	< 0.001
Listing Status 1A	2.07 (1.64–2.63)	< 0.001

Listing Status 1A was also strongly associated with the composite outcome, though not as strongly as waitlist inactivation (SHR 2.07 [1.64–2.63], *p* < 0.001). When accounting for diagnosis as a covariate, this association persisted. Using the diagnosis of cardiomyopathy as a reference, the diagnoses of congenital heart disease and “other” were both associated with an increased risk of the composite outcome (SHR 1.86 [1.46–2.36], *p* < 0.001; and SHR 3.98 [2.09–7.56], *p* < 0.001, respectively).

**FIGURE 4:**
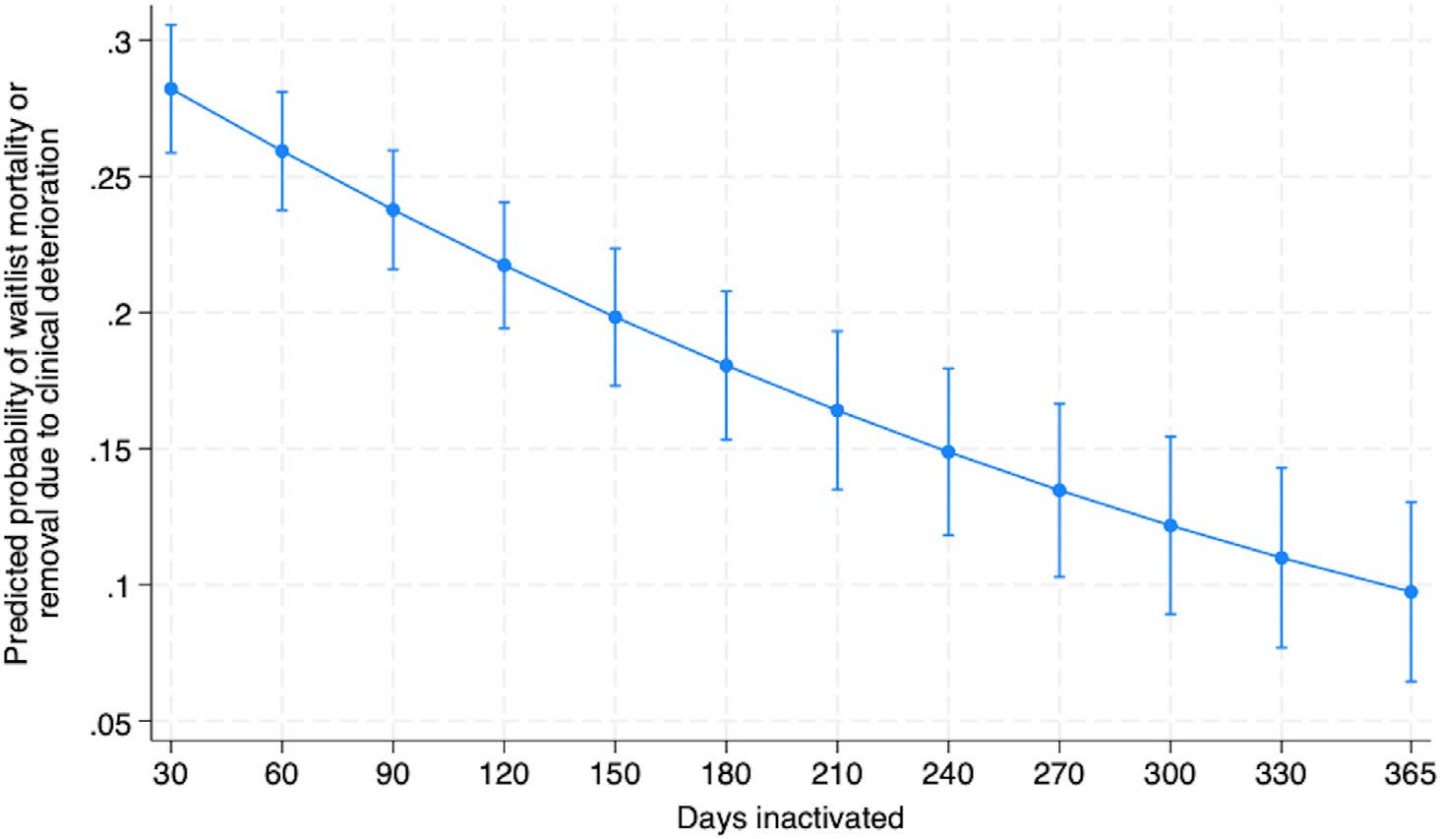
Adjusted predictions for the composite outcome of waitlist mortality or removal due to clinical deterioration as a function of inactive time with 95% confidence intervals.

Logistic regression with estimated marginal means was then employed to predict the composite outcome of waitlist mortality or removal due to clinical deterioration as a function of inactive time on the waitlist. Figure 4 shows that the predicted probability of the composite outcome decreased steadily with longer inactive time, falling from 28.2% at 30 days of inactive time to 9.9% at 1 year of inactive time.

### 3.4 Posttransplant Survival

**FIGURE 5:**
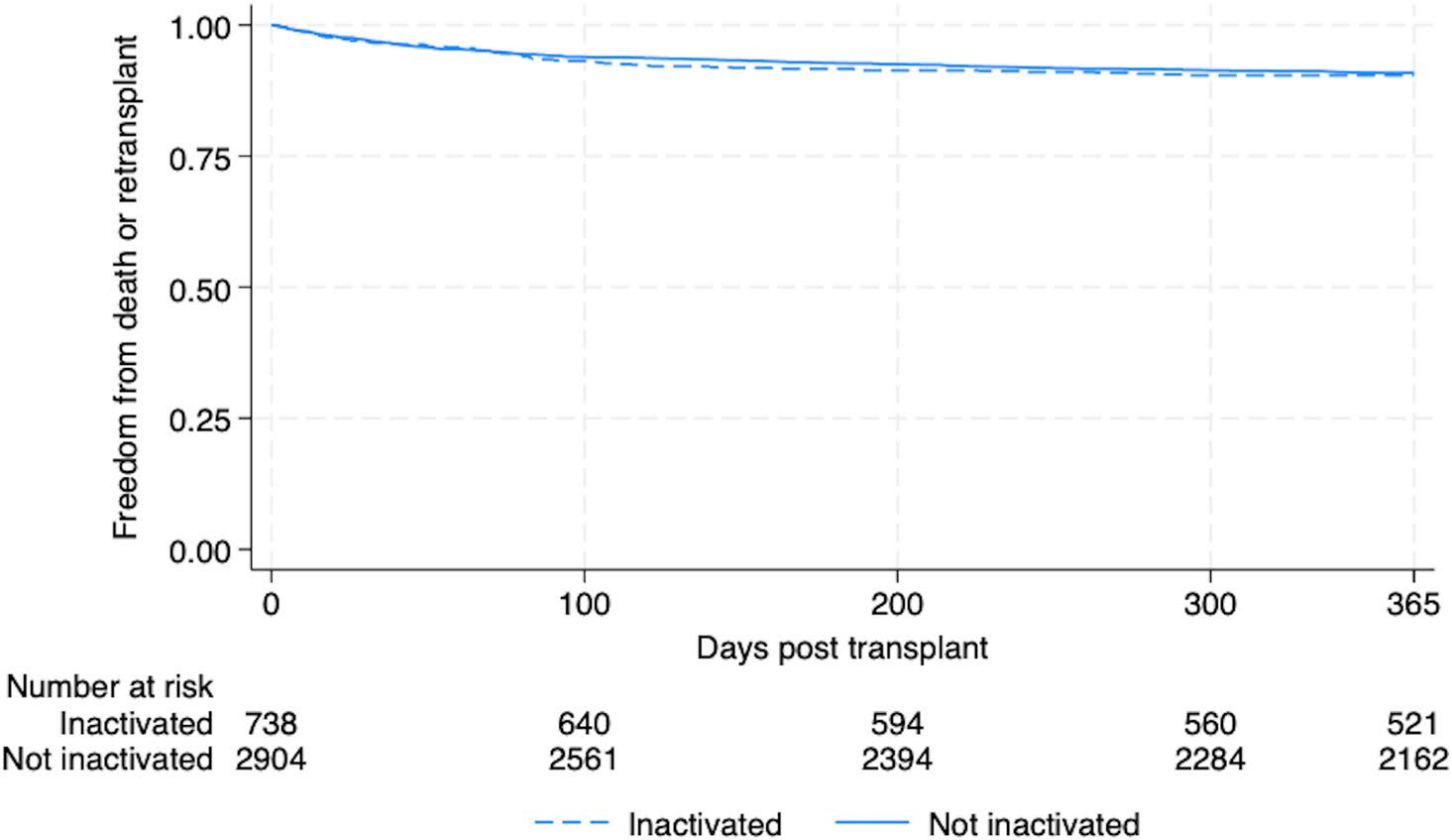
Kaplan–Meier analysis of posttransplant survival at 1 year.

Lastly, Kaplan–Meier analysis was employed to compare short‐term posttransplant survival between inactivated and non‐inactivated patients (Figure 5). Survival at 1 year following heart transplant was similar between groups (*p* = 0.889).

## 4 Discussion

In this OPTN database study, we profiled pediatric heart transplant candidates and recipients with a history of waitlist inactivation and compared their characteristics to candidates who were not inactivated. We found that inactivation is common, mostly due to temporary illness but reasons for inactivation varied by age. Inactivated patients had longer waitlist duration as well as higher waitlist mortality—particularly due to extracardiac causes—though the risk of waitlist mortality decreased with longer inactive time. Inactivation made no difference in short‐term posttransplant outcomes.

In other studies of pediatric heart transplant waitlist mortality, a common finding is that waitlist mortality is higher for candidates listed at higher urgency statuses, though there seems to be inefficiency in the current donor heart allocation system as approximately two‐thirds of candidates are listed as Status 1A. Moreover, not all candidates who qualify for listing as Status 1A carry the same risk of waitlist mortality [3, 4]. Our findings are important as they are the first to explore the influence of waitlist inactivation on waitlist and posttransplant outcomes in the current allocation era. In doing so, our study also revealed several interesting themes characterizing pediatric heart transplant listing practices.

We found that having a history of waitlist inactivation was associated with a longer total waitlist duration. At first glance, this extended waitlist duration might be assumed to be related to prolonged illness, especially given the increased risk of waitlist mortality associated with inactivation. However, waitlist inactivation encompasses a broad range of clinical contexts with different prognostic implications. This loose operational definition of “inactivation” is highlighted in the fact that those who were inactivated for temporary illness had the shortest inactive time. Conversely, those who were inactivated for clinical improvement had the longest inactivated time, suggesting that there are a significant number of patients who remain on the waitlist while clinically improved or perhaps due to issues related to readiness. This interpretation is further supported by the waitlist disposition of those inactivated for clinical improvement and readiness, and by the fact that there was no significant association between diagnosis and reason for inactivation. Even patients with a history of inactivation who eventually reached transplant mostly did so in the outpatient setting, again suggesting that longer waitlist duration and inactive time do not necessarily indicate sicker clinical status.

Our model for the probability of adverse waitlist outcomes according to inactive time shows that the probability of waitlist mortality or removal due to clinical deterioration decreases with longer inactive time. This model supports the notion that nonclinical factors (such as a reluctance to proceed with waitlist removal after clinical improvement or logistical challenges related to transplant readiness) may influence inactive time, waitlist duration, and waitlist mortality. Since listing practices vary between institutions, it is likely that survivorship, selection, and time‐dependent effects play a role in the model, but that may be the very point; those surviving despite longer inactive times represent a clinically distinct subgroup that survives longer because of the timing of their listing or inactivation for nonclinical reasons. Therefore, it is important not to apply the increased risk of adverse waitlist outcomes to all heart transplant candidates potentially needing inactivation.

Our data also suggest that age influences waitlist inactivation. While younger patients were inactivated more frequently overall and particularly for clinical reasons, older patients were inactivated more often due to readiness issues. We postulate that the reason for this difference may be due to the longer waitlist times required for younger patients. If younger patients are less likely to receive a donor offer early in their waitlist time, then clinicians may be less likely to inactivate them for temporary nonclinical reasons as a strategy to accrue more waitlist time and bring them closer to transplant. On the other hand, older patients more often face competing nonclinical pressures that affect their readiness for transplant, including insurance challenges, logistical constraints such as travel, or even psychological preparedness. The pediatric listing advantage may play a role in these observations as well. We observed that 17‐year‐old patients comprised the slight majority of patients initially listed for transplant as Status 7, supporting the notion that some clinicians might be eager to list pediatric patients as they approach adult listing age to capitalize on the pediatric advantage, even if the urgency for transplant is low. Clinicians may list these older patients and quickly inactivate them, or perhaps even list them initially as Status 7, later hesitating to delist the patient if they improve but a future transplant remains possible. Therefore, they remain inactivated on the waitlist for extended periods. Indeed, in our cohort, the upper tenth percentile of patients (approximately 200 patients) inactivated due to clinical improvement had inactive times exceeding 1 year but reaching as high as 7.8 years. Nevertheless, while these listing strategies are plausible explanations for our findings, they may extend beyond what can be directly inferred from OPTN data.

Interestingly, although inactivated pediatric heart transplant candidates experienced higher waitlist mortality, there was no difference in survival at 1 year following transplant between non‐inactivated patients. These findings are similar to 3‐year posttransplant mortality observed by Goel et al. [5] prior to the 2016 pediatric heart allocation policy revision, though it is interesting to note that the frequency of inactivation was lower than in our cohort at 11%. Although the impact of waitlist inactivation on posttransplant outcomes has not been studied in the current era of pediatric heart allocation, the one published study of this relationship in adult heart transplant recipients showed different results. In the study by Briasoulis et al., the incidence of all‐cause mortality at 1 year following transplant was higher among patients with a history of waitlist inactivation. Among their cohort, waitlist inactivation was less common at 18.0%, though the proportion of inactivated patients due to illness was similar at 65.9%. In contrast to our findings, patients listed with higher urgency were inactivated more frequently [2]. The differences between their findings and ours can largely be explained by vastly different patient populations; their population was limited to adult heart transplant recipients previously listed as Status 1 and Status 2 only. In other words, their cohort was partly composed of patients bridged to transplant on ECMO or non‐dischargeable VAD support, potentially with complications. These patients have been shown to have worse posttransplant survival, even in the pediatric population [6, 7, 8].

Our findings should be interpreted in the context of several limitations. First, approximately 30% of listings in the study cohort were not associated with an anonymized transplant center in the registry, limiting interpretation of inactivation practices by transplant center volume. Second, and perhaps more important in this database study, larger study populations were achieved at the expense of valuable clinical details. For example, this limitation is relevant to our finding that inactivated patients were more often supported by VAD at listing. Transplant candidates who undergo VAD implant may be reactivated at differing times depending on center practice, and others may only be listed for transplant after successful healing following VAD implant. Therefore, inactivation could reflect a spectrum of true clinical deterioration on the waitlist or simply routine listing practice. The same lack of granular detail prevents us from tracking the course of patients over time. For example, since the database only afforded us the ability to track total inactivated time, we were unable to determine whether patients were inactivated more than once and if the number of inactivations had any influence on waitlist or posttransplant outcomes. This limitation may be particularly important because patients experiencing shorter, repeated inactivations may be quite different from patients with one prolonged inactive period. Having more data surrounding the specific timing of inactivation would be useful in determining whether inactivated patients were eventually reactivated at higher or lower urgency statuses, or if the duration of inactivation had any impact on the chance of reactivation. Since we found that the risk of waitlist mortality is low for patients with longer inactive time, we conjecture that these patients are eventually removed, though we do not have the longitudinal data to assess this in all patients with certainty. All of these issues unfortunately cannot be answered with the level of detail and undocumented confounding variables in a database study.

Despite these limitations, our findings are still impactful because they offer insight into waitlist practices that may ultimately inform pediatric donor heart allocation policy. Several reports call for a more nuanced donor heart allocation policy that takes more clinical details into account [3, 4, 9, 10]. Our findings support this notion to further risk stratify patients, perhaps using more dynamic factors—such as a recent history of inactivation—as a marker of increased risk of waitlist mortality. Another consideration would be to include cause‐specific inactivation in the calculation of risk, as a patient who is inactivated for clinical improvement or transplant readiness issues would not have the same risk of waitlist mortality as a patient inactivated for temporary illness. Ultimately, waitlist inactivation is not a uniformly high‐risk state; rather, the prognostic implications of waitlist inactivation appear heterogeneous and dynamic over time. Continuous distribution is a developing organ allocation policy that is well positioned to consider these heterogeneous effects of inactivation on waitlist outcomes.

## Funding

The authors have nothing to report.

## Conflicts of Interest

The authors declare no conflicts of interest.

## Supporting information


**Table S1:** Patterns of inactivation by listing region.
**Table S2:** Patterns of inactivation by transplant center volume (*n* = 3666).

## Data Availability

The data that support the findings of this study are openly available in the Organ Procurement and Transplantation Network Database at https://hrsa.unos.org/DataRequestInstructions.

## References

[1] Organ Procurement & Transplantation Network , “OPTN Policy,” accessed February 3, 2025, https://optn.transplant.hrsa.gov/policies‐bylaws/policies/.

[2] A. Briasoulis , T. Rempakos , I. Doulamis , I. P. Doulamis , and P. Alvarez , “Prognostic Implications of Inactive Status in Highest Urgency Categories Among Heart Transplantation Recipients in the New Donor Heart Allocation System,” Clinical Transplantation 37, no. 2 (2023): e14861, 10.1111/ctr.14861. 36394372

[3] A. Power , K. Sweat , A. Roth , et al., “Contemporary Pediatric Heart Transplant Waitlist Mortality,” Journal of the American College of Cardiology 84, no. 7 (2024): 620–632, 10.1016/j.jacc.2024.05.049. 39111968

[4] R. J. Butts , L. Toombs , J. K. Kirklin , et al., “Waitlist Outcomes for Pediatric Heart Transplantation in the Current Era: An Analysis of the Pediatric Heart Transplant Society Database,” Circulation 150, no. 5 (2024): 362–373, 10.1161/CIRCULATIONAHA.123.068189. 38939965

[5] A. N. Goel , A. Iyengar , K. O. Schowengerdt , A. Fiore , and C. Huddleston , “Temporary Inactivation From the Waitlist due to Decline in Health Does Not Affect Mid‐Term Outcomes Among Pediatric Heart Transplant Recipients,” Journal of Heart and Lung Transplantation 35, no. 4S (2016): S404.

[6] B. B. Das , J. Trivedi , S. R. Desphande , et al., “Recent Era Outcomes of Mechanical Circulatory Support in Children With Congenital Heart Disease as a Bridge to Heart Transplantation,” ASAIO Journal 68, no. 3 (2022): 432–439, 10.1097/MAT.0000000000001468. 35213887

[7] J. B. Edelson , Y. Huang , H. Griffis , et al., “The Influence of Mechanical Circulatory Support on Post‐Transplant Outcomes in Pediatric Patients: A Multicenter Study From the International Society for Heart and Lung Transplantation (ISHLT) Registry,” Journal of Heart and Lung Transplantation 40, no. 11 (2021): 1443–1453, 10.1016/j.healun.2021.06.003. 34253457

[8] J. Conway , R. Cantor , D. Koehl , et al., “Survival After Heart Transplant Listing for Infants on Mechanical Circulatory Support,” Journal of the American Heart Association 9 (2020): e011890, 10.1161/JAHA.118.011890. 33076747 PMC7763397

[9] K. K. Khush , A. T. Sandhu , and W. F. Parker , “How to Make the Transplantation Allocation System Better,” JACC Heart Failure 11, no. 5 (2023): 516–519, 10.1016/j.jchf.2022.11.029. 37137658 PMC10790721

[10] P. Q. Thuan , C. D. Khang , and N. H. Dinh , “Improving the Prioritization of Heart Transplantation Candidates for Optimal Clinical Outcomes: A Narrative Review,” Current Cardiology Reports 27, no. 8 (2025): 1–14, 10.1007/s11886-024-02150-2. 39777580

