# Supplementary material for: Effect of Pediatric Heart Transplant Waitlist Inactivation on Waitlist and Posttransplant Outcomes
Source: Pediatr Transplant. 2026 Sep 15;30(9):e70454. doi: 10.1111/petr.70454 (PMC13575578; doi:10.1111/petr.70454)
Supplement: Supplementary file 1 [file PETR-30-e70454-s001.docx]

**Effect of Pediatric Heart Transplant Waitlist Inactivation on Waitlist and Post-transplant Outcomes**

**Dean S. Karahalios et al**

**Supplementary Materials**

**Supplementary Table 1: Patterns of inactivation by listing region**

| **n (%) or median (IQR)** | **Region 1**  **(n=123)** | **Region 2**  **(n=389)** | **Region 3**  **(n=686)** | **Region 4**  **(n=562)** | **Region 5**  **(n=808)** | **Region 6**  **(n=156)** | **Region 7**  **(n=511)** | **Region 8**  **(n=475)** | **Region 9**  **(n=303)** | **Region 10**  **(n=468)** | **Region 11**  **(n=721)** | **p value** |
| --- | --- | --- | --- | --- | --- | --- | --- | --- | --- | --- | --- | --- |
| **History of inactivation** |  |  |  |  |  |  |  |  |  |  |  | <0.001 |
| **Inactivated** | 50 (40.7%) | 115 (29.6%) | 222 (32.4%) | 243 (43.2%) | 284 (35.2%) | 47 (30.1%) | 163 (31.9%) | 185 (39.0%) | 45 (14.9%) | 210 (44.9%) | 152 (21.1%) |  |
| **Not inactivated** | 73 (59.4%) | 274 (70.4%) | 464 (67.6%) | 319 (56.8%) | 524 (64.9%) | 109 (69.9%) | 348 (68.1%) | 290 (61.1%) | 258 (85.2%) | 258 (55.1%) | 569 (78.9%) |  |
| **Inactivation reason** |  |  |  |  |  |  |  |  |  |  |  | <0.001 |
| **Illness** | 23 (46.0%) | 59 (51.3%) | 119 (53.6%) | 150 (61.7%) | 175 (61.6%) | 26 (55.3%) | 99 (60.7%) | 114 (61.6%) | 22 (48.9%) | 146 (69.5%) | 59 (38.8%) |  |
| **Improvement** | 9 (18.0%) | 33 (28.7%) | 53 (23.9%) | 44 (18.1%) | 41 (14.4%) | 9 (19.2%) | 37 (22.7%) | 36 (19.5%) | 12 (26.7%) | 31 (14.8%) | 47 (30.9%) |  |
| **Readiness** | 18 (36.0%) | 23 (20.0%) | 50 (22.5%) | 49 (20.2%) | 68 (23.9%) | 12 (25.5%) | 27 (16.6%) | 35 (18.9%) | 11 (24.4%) | 33 (15.7%) | 46 (30.3%) |  |
| **Status at listing** |  |  |  |  |  |  |  |  |  |  |  | 0.008 |
| **Status 7** | 1  (0.8%) | 5  (1.3%) | 3  (0.4%) | 16 (2.9%) | 15 (1.9%) | 1 (0.64%) | 2  (0.4%) | 6  (1.3%) | 1  (0.3%) | 7  (1.5%) | 14 (1.9%) |  |
| **Status 1A, 1B, or 2** | 122 (99.2%) | 384 (98.7%) | 683 (99.6%) | 546 (97.2%) | 793 (98.1%) | 155 (99.4%) | 509 (99.6%) | 469 (98.7%) | 302 (99.7%) | 461 (98.5%) | 707 (98.1%) |  |
| **Total inactive time (days)** | 20  (8-67) | 24  (8-148) | 31  (10-98) | 33  (7-149) | 21  (6-96) | 29  (5-73) | 23  (8-294) | 21  (6-85) | 32  (6-217) | 25  (7-74) | 25  (9-198) | 0.093 |

**Supplementary Table 2: Patterns of inactivation by transplant center volume (n = 3666)**

| **n (%) or median (IQR)** | **Listings at**  **high volume center**  **(n = 974)** | **Listings at**  **medium volume center**  **(n = 2248)** | **Listings at**  **low volume center**  **(n = 444)** | **p value** |
| --- | --- | --- | --- | --- |
| **History of inactivation** |  |  |  | 0.024 |
| **Inactivated** | 222 (22.8%) | 422 (18.8%) | 96 (21.6%) |  |
| **Not inactivated** | 752 (77.2%) | 1826 (81.2%) | 348 (78.4%) |  |
| **Inactivation reason** |  |  |  | 0.015 |
| **Illness** | 163 (73.4%) | 269 (63.7%) | 74 (77.1%) |  |
| **Improvement** | 5 (2.3%) | 20 (4.7%) | 5 (5.2%) |  |
| **Readiness** | 54 (24.3%) | 133 (31.5%) | 17 (17.7%) |  |
| **Status at listing** |  |  |  | 0.038 |
| **Status 7** | 16 (1.6%) | 19 (0.9%) | 9 (2.0%) |  |
| **Status 1A, 1B, or 2** | 958 (98.4%) | 2229 (99.2%) | 435 (98.0%) |  |
| **Total inactive time (days)** | 16 (6-48) | 13 (6-33) | 21 (7-52) | 0.091 |
